# Biobanking in a Constantly Developing Medical World

**DOI:** 10.1155/2013/343275

**Published:** 2013-09-23

**Authors:** Stefan-Alexandru Artene, Marius Eugen Ciurea, Stefana Oana Purcaru, Daniela Elise Tache, Ligia Gabriela Tataranu, Mihaela Lupu, Anica Dricu

**Affiliations:** ^1^University of Medicine and Pharmacy of Craiova, Romania; ^2^Bagdasar-Arseni Hospital, Bucharest, Romania; ^3^Medico Science SRL, Craiova, Romania

## Abstract

Biobank is a very sophisticated system that consists of a programmed storage of biological material and corresponding data. Biobanks are created to be used in medical research, in clinical and translational medicine, and in healthcare. In the past 20 years, a large number of biobanks have been set up around the world, to support the modern research directions in medicine such as omix and personalized medicine. More recently, embryonic and adult stem cell banks have been developed. Stem cell banking was reported to be required for medical research as well as clinical transplant applications. The quality of the samples stored in a biobank is very important. The standardization is also important; the biological material stored in a biobank must be processed in a manner that allows compatibility with other biobanks that preserve samples in the same field. In this paper, we review some issues related to biobanks purposes, quality, harmonization, and their financial and ethical aspects.

## 1. Introduction

Biomaterial resources such as tissues, cells, blood, and serum have played a critical role in academic research. Until recently, medical research activity was based on biomaterial resources created by single investigators or research groups. Clinical trials and epidemiological studies have contributed for quite a long time to a better understanding of certain diseases, but the incredibly rapid pace at which biotechnology, medical research, and high quantities of phenotypic information which are constantly added to each patient's case demand that we take a step back and look at the bigger picture involved. Even if biobanks are regarded as a relatively new notion, the basic concept behind this stretches further down in medical history and originates in the ever expanding need to understand the clinical and epidemiological sides of different diseases and the ramifications these aspects would pose in further development of more efficient ways to cure and prevent those afflictions [1]. 

The core principle behind the birth and evolution of biobanks is the necessity to somehow centralize and facilitate the flow of information required by a more complex approach in today's research which has outpaced the traditional hypothesis-driven fundamental structure. Further on, in the paper we will discuss the various aspects of biobanking that need to be tackled in order to obtain successful structure from different points of view. 

By definition, “biobank” is a long-term storage and conservation facility for biological specimens, to support future scientific investigation [2]. Biobanks consist of two different parts: (I) the biologic material that is collected, processed, and long-time stored and (II) the database, including information about demographic and clinical data for each sample and also associated with the bank inventory.

Now a days, biobank system offers a large number of archived biospecimens, linked to clinical and molecular data that support research and clinical partnerships. Data from the literature indicates that a biobank is available in every continent [3].

The main activities of a biorepository are biospecimens collection, processing, storage, or inventory and distribution of biological material.

**Figure 1:**
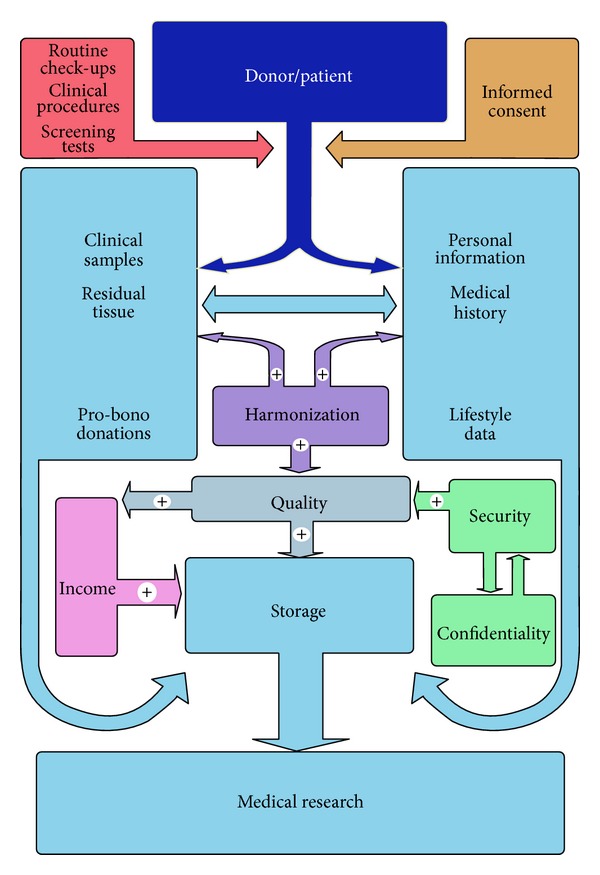
Overview of the circuit of data and samples within a biobank. Blue colors represent information and materials circulating from the donor/patient through different steps while the other colors represent various factors, which influence the status of the biobank. The biobanks are different, having different characteristics, depending on the biological material they store (i.e., tissues, cells, blood, biological fluids, DNA, RNA, etc.), the types of information they collect, and also how the data is processed and organized [4].

Thus, biospecimen sample collection and processing is followed by recording of information about the sample. In general, the information that accompanies the sample includes samples source, characteristics, postcollection processing, and storage. An example of a biobank for medical research is shown in Figure 1.

The cooperation between different institutions is the key to a more robust, more efficient, and also more flexible structure which allows information and biological samples to be pooled, analyzed, and shared between the aforementioned singular biobanks. Therefore, the biobanks should harmonize their procedures for collecting, storing, and agreement in order to maximize sharing (Figure 1).

The quality of stored biomaterial quality is also a key factor in achieving a successful biobank. Therefore, working protocols should include clear and detailed instructions for collecting, processing, and storing of the biological material (Figure 1).

Depending on the purpose and reason, there are several types of biobanks: disease oriented biobanks, population based biobanks, Case-control biobanks, tissue biobanks, biobanking within the context of clinical trials, biomolecular resource centres (antibodies, etc.), biobanks for cells (cord blood, stem cells), and so forth [5]. 

Due to the enormous amount of information contained in the samples, biobanks are an important reserve in development and validation of new diagnostic markers and new therapeutic agents. In cancer research, biobanks are a key resource for genomic-, proteomic-, and metabolomic-based research, molecular epidemiology and translational studies, molecular diagnostic and therapy, development of therapeutic targets, and biomarker and drug discovery.

The ability of embryonic and adult stem cells to give rise to any cell tissue type (hematopoietic and mesenchymal lineages) has led to enormous interest in their use in translational and clinical approaches. 

Therefore, stem cell biobanks have received much attention as a new biologic resource for medical research as well as clinical transplant applications. In the last 10 years, embryonic and adult stem cell banks have been developed around the world [6]. 

Recently, induced pluripotent stem cells (iPSCs) banks have also emerged as other potential sources for medical research and clinical applications [7]. 

Currently, iPSCs derived from adult human somatic cells are used in several medical research areas (drug development, drug screening, disease models, etc.). In addition, iPSCs opened a new field of stem cell based therapeutic strategies for a large number of human diseases such as neurological diseases [8], heart disorder [9], liver diseases [10], diabetes [11].

## 2. Quality 

A key structural pylon on which the biobank is being built and further evolved is, unquestionably, quality. This encompasses a very wide range of variables on which the overall value of the information contained within the biobank is rated [12]. Firstly, we have to take into consideration the preanalytical phase of the collection of samples which is split into two phases: the preacquisition phase which takes place before the actual contact with the biobank personnel and the acquisition phase when these samples come under the supervision and their value is assessed by the biobank personnel. During the preacquisition phase a number of circumstances have to be considered. For example, it is of great importance to know if the donor was under any form of treatment with drugs such as antibiotics, anticoagulants, anesthetics, or pretreatments [13]. Another quality indicator during the preacquisition phase is lag time, the time between the removals of the specimen from the body till it is frozen [14]. This circumstantial factor is of great importance to those involved in biobanking and/or research; however, it frequently presents little to no interest to surgeons who are under a lot of pressure during a high-risk operation, for example. After all these have been taken into consideration, another important variable is in regard to the acquisition phase of the process: the nature and the quality of the storage of the sample [15]. It is advisable, when possible, for tissue samples to be stored in different formats (e.g., blood: whole blood, plasma, and serum) and also in accordance to present international guides of conduct in order to facilitate the implementation of modern technological processes through a wider array of samples which in turn, of course, adds up to the value of those specific samples [16].

In the process of sample banking, it is very important to record and track each step from collection through storage. Inadequate sample preparation can lead to inexact results, difficulties in interpretation, and incorrect assessment. Therefore, working protocols should include clear and detailed instructions for collecting, processing, and storing of the biological material [17]. 

The sheer value of the samples in the biobank is not only defined by just its physical qualities but also by the abundance and the quality of the data associated with it. Proper annotation must include crucial information such as treatments and outcome of each treatment, diagnosis, time of death, and correct pathological traits, [18]. Incomplete or, even worse, incorrect data can lead to samples with little value and even may, as a worst case scenario, influence the outcome of a study. 

## 3. Harmonization 

Typically, the collection of samples required for a study can take years, even decades in the case of studies related to rare diseases. This large interval of time can negatively influence the desired result of the study, due to a constant appearance of new scientific insights which can alter both the procedures involved in the experiments and even the end result of the whole study. 

Harmonization is an essential step in overcoming this inconvenience, and it involves mutually beneficial cooperation between different biobanks and networks [19]. 

In addition, the collection, processing, and storage procedures are different from one to another biobank, limiting the collaboration between research groups [20]. 

There is a huge number of international literature on procedures and guidelines for biobanks [21, 22]. 

The U.S. Food and Drug Administration (FDA), the U.S. Centers for Disease Control (CDC), and professional organizations, such as the American Association of Tissue Banks (AATB), the National Cancer Institute (NCI), and the International Society for Biological and Environmental Repositories (ISBER), the Organisation for Economic Co-operation and Development (OECD), and the International Agency for Research on Cancer (IARC), provide guidelines for biorepositories.

However, at this time there is a short list of organizations and entities that are successful in sharing common harmonized protocols: (1) regional and international organizations: IARC, FIBO, and ESBB, (2) science and infrastructure initiatives: BBMRI, caHUB, caBIG, The Biomarkers Consortium, BioSHaRE-EU, HuGENET, and PHOEBE, (3) research tools and databases: dbGap, datSHaPER, dataSHIELD, HapMap, HUMGEN, OBIBA, and OBO. 

More biobanks should therefore harmonize their procedures for collecting, storing, and agreement in order to maximize sharing.

This interoperability between different entities is the key to a more robust, more efficient, and also more flexible structure which allows information and biological samples to be pooled, analyzed, and shared between the aforementioned singular biobanks. The key to a successful harmonization resides in a few common denominators regarding the standardization of the protocols involved in managing each biobank. These include specific ontology, technological procedures concerning sample storage and handling, software used for analytical tools, algorithms and data processing, common exchange formats, and last but not least, compatible ethico-legal frameworks [23]. 

The end result can be viewed as organizations or groups presided by a common interest which is a wider, more accessible network of biosamples, crucial data related to these samples, and a better integration of both clinical and nonclinical research information. The beneficiaries of these networks are the scientific communities which gain access to higher, better quality and more complex amounts of information which are homogenously gathered, stored, and annotated under specific standards operating procedures [24]. 

## 4. Financial Aspects 

Probably, one of the hardest aspects to handle is the financial side of biobanking. This stems from multiple reasons such as funding difficulties, adapting to the constantly changing market needs, creating a compatible business model on which to attain long-term sustainability, cost and recovery. 

It is no secret that the economic climate, as of late, has been turbulent. In this unsteady environment, large entities with the potential to invest capital into a project like a biobank (e.g., pharmaceutical companies, governments or nonprofit organizations) are scarce. This reluctance is based on the fact that just the start-up investment for such a project requires a huge amount of capital redirected towards infrastructure, operating costs, sample acquirement, personnel, and costs which are included into a business model based on limited experience and scarce data. Simply put, biobanking still represents a young medical sector, and models on which to build a value proposition to be presented to possible investors are, indeed, very limited [25]. Also, in order to yield satisfying results, a biobank must be constructed based on a long-term commitment policy, stretching on for years, even decades. Adding these up it is reasonable to understand why private or public investors are not so eager to invest capital into such a project. 

After its creation, in order to survive, a biobank must concentrate on fulfilling the demands of the market for human samples used for various research purposes and preclinical drug trials. This is done by concentrating on the acquisition and storage of samples of both high quality and great demand from the scientific community based on reports from investment firms associated with pharmaceutical companies and also having reliable information regarding progress within the academic community [26]. It is always a good idea to keep an ear to the ground, as some might say. In turn, having a high demand for samples adds up to the licensing fees demanded by the biobank and also generates good reputation. Likewise, failure to comply with the market demand will result in a large number of samples of little value, stored within the facility, generating unwanted extra costs for maintenance and will decrease the total value of the information contained within the bank.

 Licensing biospecimens and other sources, such as custodial collection services, genotyping, trainings on the management of biobanks and biospecimens, molecular and proteomic analysis, will become cost recovery and coupled with public or private funding will balance out the costs for biospecimen collecting and shipping, biospecimen processing, storage management and the retrieval and distribution of the samples (Biobanking value chain framework) [27]. Adjust these numbers to a yearly inflation rate, value depreciation over time, and add a risk margin for unexpected circumstances, and you will have a sustainable and durable business model which will support a long-term project [28]. 

## 5. Ethical Aspects 

The issue of ethics in biobanks revolves around multiple highlights which have to be addressed in order to correctly manage the legal and, more importantly, the moral aspects of this line of work. 

The core of this issue is informed consent. Informed consent derives from the golden standard that every donor/patient has the right of self-determination in regard to the samples included in the biobank [29]. This means that before any action takes place that person must be informed to a full extent about the nature of biobanking, the line of work in which the samples he has donated might be involved in, and, very importantly, how the outcome of the research, to which his samples have been subjected, might affect him in the future [30]. 

The methods through which informed consent can be obtained are either the opt-in or opt-out options [31]. Opt-in requires the express written consent of the donor in which he agrees to participate in future research while opt-out translates the inaction of the donor, after being informed about the implications of his agreement, as a sign of consent. Each option has its pros and cons which make both of them viable, but a consensus regarding which one is superior has not been reached. The opt-in variant presents respect for negative autonomy, encourages a higher degree of positive autonomy, promotes scientific citizenship, offers stronger legal coverage for the researchers involved, and projects a better image perceived by the public eye [32]. On the other hand, the opt-out option involves higher participation rates, lower costs because less money is spent chasing each person's consent, compatibility of autonomy given that each participant has been thoroughly informed about the implications of the research beforehand, and also a general positive public attitude in regard to the inclusion of certain residual tissue in future research [33]. Choosing one of these methods is up to the biobank personnel which also have to take into consideration aspects such as religious beliefs of those involved, certain national or federal laws which may affect their decision, and public opinion at the time. 

Other issues which have to be addressed are confidentiality and the protection of the information associated with each sample [34]. One cannot stress enough on the importance of confidentiality when handling sensitive information regarding the patient. The breach of this confidentiality could subject the aforementioned donor to stigmatization, discrimination, and other forms of harassment, implications which clearly he does not desire when accepting to participate in the structures of a biobank. Security of information within the biobank is also of great importance due to the valuable nature of some of the data stored within [35]. Inadequate security measures could lead to third parties having direct access to sensitive data which in turn can be used for different purposes such as selling this information for an illicit profit to commercial entities, providing private medical records to employers regarding their employees, or even blackmailing certain individuals. 

## 6. Conclusion

Advances in epidemiology and omix science have led to an increased interest in infrastructure development and data sharing facilitated by biobank of specimens and linked health information. 

In medical environment, biobanks are a key resource for genomic-, proteomic-, and metabolomic-based research, molecular epidemiology and translational studies, molecular diagnostic and therapy, development of therapeutic targets and biomarker and drug discovery. Therefore, it is not surprising that in the recent years the increasing interest in biobanking has been demonstrated by both academic and industrial researchers.

## References

[1] Organisation for Economic Co-operation and Development (OECD) (2001). *Biological Resource Centres: Underpinning the Future of Life Sciences and Biotechnology*.

[2] Council of Europe (2006). *Recommendation Rec 4 of the Committee of Ministers to Member States on Research on Biological Materials of Human Origin*.

[3] Melsin EM, Goodman K (2009). *Biobanks and Electronic Health Records: Ethical and Policy Challenges in the Genomic Age*.

[4] Yuille M, Dixon K, Platt A (2010). The UK DNA banking network: a “fair access” biobank. *Cell and Tissue Banking*.

[5] http://www.coe.int/t/dg3/healthbioethic/Activities/10_Biobanks/biobanks_for_Europe.pdf.

[6] Goh BC, Thirumala S, Kilroy G, Devireddy RV, Gimble JM (2007). Cryopreservation characteristics of adipose-derived stem cells: maintenance of differentiation potential and viability. *Journal of tissue engineering and regenerative medicine*.

[7] Gourraud P, Gilson L, Girard M, Peschanski M (2012). The role of human leukocyte antigen matching in the development of multiethnic “haplobank” of induced pluripotent stem cell lines. *Stem Cells*.

[8] Inoue H (2010). Neurodegenerative disease-specific induced pluripotent stem cell research. *Experimental Cell Research*.

[9] Terrenoire C, Wang K, Tung KWC (2012). Induced pluripotent stem cells used to reveal drug actions in a long QT syndrome family with complex genetics. *The Journal of General Physiology*.

[10] Chun YS, Chaudhari P, Jang Y (2010). Applications of patient-specific induced pluripotent stem cells; focused on disease modeling, drug screening and therapeutic potentials for liver disease. *International Journal of Biological Sciences*.

[11] Kelly C, Flatt CCS, McClenaghann NH (2011). Stem cell-based approaches for the treatment of diabetes. *Stem Cells International*.

[12] National Cancer Institute Best practices for biospecimen resources. http://biospecimens.cancer.gov/global/pdfs/NCI_Best_Practices_060507.

[13] McCarty CA, Wilke RA (2010). Biobanking and pharmacogenomics. *Pharmacogenomics*.

[14] Spruessel A, Steimann G, Jung M (2004). Tissue ischemia time affects gene and protein expression patterns within minutes following surgical tumor excision. *BioTechniques*.

[15] Womack C, Gray NM (2009). Banking human tissue for research: vision to reality. *Cell and Tissue Banking*.

[16] Mager SR, Oomen MHA, Morente MM (2007). Standard operating procedure for the collection of fresh frozen tissue samples. *European Journal of Cancer*.

[17] NCI (2007). *Best Practices for Specimen Resources*.

[18] International Society for Biological and Environmental Repositories (ISBER) (2008). Best practices for repositories: collection, storage, retrieval and distribution of biological materials for research. *Cell Preservation Technology*.

[19] Riegman PHJ, Dinjens WNM, Oomen MHA (2006). TuBaFrost 1: uniting local frozen tumor banks into a European network, an overview. *European Journal of Cancer*.

[20] The Wellcome Trust (2011). *Sharing Research Data to Improve Public Health: Full Joint Statement by Funders of Health Research*.

[21] Hansson MG, Dillner J, Bartram CR, Carlson JA, Helgesson G (2006). Should donors be allowed to give broad consent to future biobank research?. *The Lancet Oncology*.

[22] AORN (2006). Recommended practices for surgical tissue banking. *AORN Journal*.

[23] Mager R, Ratcliff C, Knox K (2004). *The NCRI Cancer Tissue Resource: Developing an Operational Framework*.

[24] Grizzle WE, Aamodt R, Clausen K, LiVolsi V, Pretlow TG, Qualman S (1998). Providing human tissues for research: how to establish a program. *Archives of Pathology and Laboratory Medicine*.

[25] Aldridge S (2005). Biobanking emerging as a key growth area. *Genetic Engineering News*.

[26] Grizzle WE, Sexton KC, Bell WC (2008). Frontiers in clinical research: quality assurance in tissue resources supporting biomedical research. *Cell Preservation Technology*.

[27] Tupasela A (2006). Locating tissue collections in tissue economies—deriving value from biomedical research. *New Genetics and Society*.

[28] Office of Management and Budget (OMB)

[29] Eriksson S, Helgesson G (2005). Potential harms, anonymization, and the right to withdraw consent to biobank research. *European Journal of Human Genetics*.

[30] Hoeyer K, Olofsson B, Mjörndal T, Lynöe N (2005). The ethics of research using biobanks: reason to question the importance attributed to informed consent. *Archives of Internal Medicine*.

[31] Maschke KJ (2006). Alternative consent approaches for biobank research. *The Lancet Oncology*.

[32] Stjernschantz FJ, Hansson MG, Eriksson S (2011). Biobank research: who benefits from individual consent?. *British Medical Journal*.

[33] Clark AM, Findlay IN (2005). Attaining adequate consent for the use of electronic patient records: an opt-out strategy to reconcile individuals’ rights and public benefit. *Public Health*.

[34] Cambon-Thomsen A, Rial-Sebbag E, Knoppers BM (2007). Trends in ethical and legal frameworks for the use of human biobanks. *European Respiratory Journal*.

[35] http://www.cai.gouv.qc.ca/indexen.html.

